# Evaluating health outcomes in the treatment of hypothyroidism

**DOI:** 10.3389/fendo.2022.1026262

**Published:** 2022-10-18

**Authors:** Matthew D. Ettleson, Maria Papaleontiou

**Affiliations:** ^1^ Section of Endocrinology, Diabetes, and Metabolism, University of Chicago, Chicago, IL, United States; ^2^ Division of Metabolism, Endocrinology and Diabetes and Institute of Gerontology, University of Michigan, Ann Arbor, MI, United States

**Keywords:** hypothyroidism, health outcome, patient-reported outcomes (PROs), health disparities, cardiovascular outcome

## Abstract

Clinical hypothyroidism is defined by the inadequate production of thyroid hormone from the thyroid gland to maintain normal organ system functions. For nearly all patients with clinical hypothyroidism, lifelong treatment with thyroid hormone replacement is required. The primary goal of treatment is to provide the appropriate daily dose of thyroid hormone to restore normal thyroid function for each individual patient. In current clinical practice, normalization of thyrotropin (TSH) level is the primary measure of effectiveness of treatment, however the use of a single biomarker to define adequate thyroid hormone replacement is being reevaluated. The assessment of clinical health outcomes and patient-reported outcomes (PROs), often within the context of intensity of treatment as defined by thyroid function tests (i.e., undertreatment, appropriate treatment, or overtreatment), may play a role in evaluating the effectiveness of treatment. The purpose of this narrative review is to summarize the prominent health outcomes literature in patients with treated hypothyroidism. To date, overall mortality, cardiovascular morbidity and mortality, bone health and cognitive function have been evaluated as endpoints in clinical outcomes studies in patients with treated hypothyroidism. More recent investigations have sought to establish the relationships between these end results and thyroid function during the treatment course. In addition to clinical event outcomes, patient-reported quality of life (QoL) has also been considered in the assessment of adequacy of hypothyroidism treatment. From a health care quality perspective, treatment of hypothyroidism should be evaluated not just on its effectiveness for the individual patients but also to the extent to which patients of different sociodemographic groups are treated equally. Ultimately, more research is needed to explore differences in health outcomes between different sociodemographic groups with hypothyroidism. Future prospective studies of treated hypothyroidism that integrate biochemical testing, PROs, and end result clinical outcomes could provide a more complete picture into the effectiveness of treatment of hypothyroidism.

## Introduction

Health outcomes research primarily aims to study the end results of health care practices and interventions. Within the scope of health care quality, health outcomes can serve as a form of measurement of the effectiveness of a specific treatment or group of treatments, in particular for chronic diseases in which the disease course is managed over time (1). Hypothyroidism is one of the most common endocrine diseases and is often managed over the course of the patient’s lifetime (2). Traditionally, the effectiveness of thyroid hormone replacement has been measured by restoration of thyroid hormone levels, especially TSH, to normal ranges. However, a growing body of evidence has demonstrated abnormal metabolic parameters (3), as well as persistent symptoms and overall patient dissatisfaction with thyroid hormone replacement in a subset of patients (4), in the face of normalized thyroid hormone levels. While TSH remains the primary biomarker for the diagnosis and management of hypothyroidism in clinical practice, additional measures for the effectiveness of thyroid hormone treatment on a population scale remain underexplored. This narrative review seeks to outline the prominent literature in health outcomes—including clinical, neurocognitive, and patient-reported outcomes—to examine the relationship between thyroid hormone replacement, thyroid function tests, and end result health outcomes. By examining hypothyroidism care through the lens of health outcomes, we can expand the definition of success in the treatment of hypothyroidism to include additional outcomes that directly reflect the patients’ longevity, morbidity, and quality of life.

## Methods

The literature included in this narrative review was identified from PubMed searches of English-language articles (1990 – present), articles previously identified by the authors from prior work, and the review of citations from the aforementioned articles. PubMed searches included the following key terms: “hypothyroidism,” “health outcomes,” “levothyroxine,” “mortality,” “cardiovascular,” “bone health,” “cognitive,” and “patient-reported outcomes.” Case series and case reports were not included. In the case when relevant review articles were identified, both the review article and the primary literature were cited. In keeping with the scope of this narrative review, literature was selected that examined end result clinical outcomes of hypothyroidism treatment that represent significant morbidity for the affected patient. Studies of clinical outcomes including all-cause mortality, cardiovascular morbidity and mortality, bone health, and neurocognitive outcomes are the focus of this review because they have been the most extensively studied and reflect end result outcomes, although studies of other clinical outcomes do exist. Additionally, only studies that directly compared treated hypothyroidism to healthy controls without thyroid disease or compared groups of treated patients with different TSH levels were included. Studies comparing patients with treated and untreated hypothyroidism (including subclinical hypothyroidism) or comparing treated subclinical hypothyroidism alone with healthy controls were excluded.

It is important to clarify that, given outcomes research often captures real-world data, a significant number of study participants treated with thyroid hormone likely presented with subclinical hypothyroidism (a condition defined by an elevated TSH level with a normal free T4 level). There are clear practice patterns that indicate that treatment with thyroid hormone is commonplace for subclinical hypothyroidism along with overt hypothyroidism (5, 6). Some studies examining health outcomes of the use of levothyroxine do not distinguish between treatment for overt and subclinical hypothyroidism, although we have assumed in such cases that the patient population includes both those treated for overt and subclinical hypothyroidism. Despite this important limitation, we have included such studies in the review because they offer important insights into the effects of overtreatment and undertreatment with thyroid hormone.

## The evolution of markers of thyroid hormone status

Prior to the widespread clinical use of TSH and thyroid hormone radioimmunoassays, physicians relied on measurable bio-metabolic parameters to determine the appropriate dose of thyroid hormone replacement for patients with hypothyroidism. These included heart and respiratory rates, weight changes, basal metabolic rate, serum protein-bound iodine level, and symptoms of hypothyroidism (undertreatment) and hyperthyroidism (overtreatment) (7, 8). During this time period, providing consistent thyroid hormone replacement was challenging due to the imprecise measurement and nonspecific nature of the above parameters, in addition to inconsistent thyroxine (T4) and triiodothyronine (T3) content within a variety of thyroid hormone preparations (9). The dual discoveries of the TSH radioimmunoassay (10) and peripheral conversion of oral T4 to T3 in humans (11) provided clinicians with a quantifiable biomarker and more consistent thyroid hormone preparations with which to treat hypothyroidism. Over time, advances in thyroid hormone preparation bioequivalence have resulted in generic forms of levothyroxine being virtually interchangeable without affecting TSH levels, going against long-held conventional wisdom that patients must stay on a single thyroid hormone preparation for as long as possible (12).

In today’s clinical practice, thyroid function tests have become the primary factors in the determination of euthyroidism in those taking thyroid hormone replacement. In hindsight, this practice shift was predictable in that it provided both physicians and patients with a clear “normal/abnormal” dichotomy regarding thyroid hormone replacement, which is desirable in a clinical environment that is burdened with growing medical complexity in the setting of clinic visit time constraints. However, there is evidence that the “normal/abnormal” dichotomy of thyroid function tests (and TSH in particular), while an essential feature of clinical practice, may not fully determine if euthyroidism has been achieved from a multi-organ system perspective for select patients (13). Data from patient surveys have demonstrated that a significant minority of patients are dissatisfied with their thyroid hormone treatment (4, 14). These results have led investigators to explore whether TSH and other thyroid function tests alone are a sufficient measure of euthyroidism (15). In addition, there is a large body of evidence from levothyroxine-treated patients that subnormal energy expenditure (16–18), abnormal lipid metabolism (3, 19, 20), unresolved cognitive impairments (21), and decreased quality of life (QoL) (22, 23) were observed concurrently with normal TSH levels. There is no single explanation for why some patients with normal serum thyroid function tests have persistent metabolic and cognitive abnormalities. It is likely that thyroid-specific and general non-specific patient factors contribute. In addition to the effects of aging and non-thyroid comorbidities, it is likely that individual differences in thyroid hormone gastrointestinal absorption (24), tissue-specific deiodinase activity (25), and hormone transport across cellular membranes (26) contribute to these abnormalities observed in some patients. In clinical practice, TSH remains the most important biomarker for the diagnosis and management of hypothyroidism. From a population perspective, the limitations of TSH and thyroid hormone levels to fully capture the thyroid status of all patients taking thyroid hormone has led to an emphasis on other measures of hypothyroidism treatment success: clinical and patient-reported health outcomes.

## Treatment of hypothyroidism and clinical health outcomes

If thyroid function testing alone does not provide a *complete* picture of the thyroid status for a significant minority of patients treated with thyroid hormone, then the evaluation of the effectiveness of thyroid hormone therapy across a population of patients should include additional outcome measures. In conjunction with thyroid function tests, evaluation of health outcomes in treated patients would be informative. From this perspective, the goal of thyroid hormone replacement would be to normalize thyroid function tests *and* allow patients with hypothyroidism to achieve health outcomes that are indistinguishable from those without thyroid disease.

### All-cause mortality

Several observational studies examining large patient populations have sought to determine the association between treated hypothyroidism and mortality. Relatively few published studies have compared all-cause mortality rates of those with treated hypothyroidism and those with normal thyroid function. In two earlier studies, all-cause mortality rates between treated hypothyroid patients and euthyroid controls were not significantly different (27, 28). Of note, TSH levels over the time course of the study were not included in either study, and those treated for subclinical hypothyroidism were likely included in the claims-based study (27). Conversely, a recent study of a large retrospective Korean claims database found all-cause mortality rates were increased in levothyroxine-treated patients (N=501,882; thyroid function tests were not reported) compared to a control cohort (N=1,505,646), with relative risk being higher in patients under 65 years of age and in those with higher cardiovascular risk at baseline (29). Similar to the previous studies, a major limitation of this study is that no thyroid function tests were collected in the study, and patients treated for overt and subclinical hypothyroidism could not be differentiated.

More recently, several studies have examined mortality relative to overtreatment and undertreatment as defined by TSH levels in different clinical settings (30–33). There is general agreement amongst these studies that overtreatment and undertreatment of hypothyroidism is associated with increased risk for mortality, thus prompting experts to reinforce the importance of the normalization of TSH levels in the treatment of hypothyroidism (34). Specifically, a retrospective cohort study of the Danish National Patient Register examining serial TSH levels in treated patients found that each six-month period of an above- or below-normal TSH level was associated with increased risk of mortality compared to controls without hypothyroidism (35). Results were similar between those treated for overt and subclinical hypothyroidism. Importantly, overtreatment and undertreatment with thyroid hormone are not consistently defined from study to study, with some relying on a single TSH value to determine level of treatment. This is a limitation of studies relying on real-world clinical data in which thyroid function tests are often collected in a sporadic manner (36).

### Cardiovascular outcomes

Thyroid function abnormalities are known to exert prominent effects on multiple organ systems, including the cardiovascular system (37). Similar to studies of all-cause mortality, several studies have examined the effect of treatment of hypothyroidism on key cardiovascular (33, 38–40) and stroke (41, 42) outcomes. Similar to mortality outcome studies, there is a general trend in the literature that overtreatment and undertreatment of hypothyroidism does increase the risk of worse cardiovascular outcomes, including stroke. To take into account variations in treatment over time, some studies have used a variety of longitudinal analytical approaches to capture changes in TSH over time when examining the association between treatment and cardiovascular outcomes (38, 39, 42). As an example, a large retrospective study of US veterans modeled cardiovascular mortality in thyroid hormone users with TSH and free thyroxine (FT4) as time-varying explanatory covariates, which provided researchers with the ability to associate specific TSH and FT4 levels with cardiovascular mortality risk levels (39). In a population of patients treated for either overt or subclinical hypothyroidism, the study demonstrated worsening cardiovascular risk the further the TSH level moved away from the reference range.

### Bone health outcomes

Thyroid hormone also exerts prominent effects on bone, both during childhood during the growth phase, and during adulthood when thyroid hormone stimulates osteoclastic bone resorption (43). Several outcome studies have examined osteoporosis and fracture risk in the setting of levothyroxine use (44–47). In a retrospective study of the Danish National Patient Registry using TSH as a time-varying covariate, overtreatment with levothyroxine was associated with increased fracture risk in women over the age of 50 (47). In a retrospective study of levothyroxine-treated individuals aged 70 years and older, those with a current prescription of levothyroxine were more likely to fracture than those whose last prescription ended over 6 months prior to fracture (46). The effect was more pronounced when the dose of levothyroxine was higher, suggesting those on higher doses of levothyroxine are at increased risk of overtreatment, although thyroid function tests were not collected in this study. In summary, overtreatment appears to be a primary driver of adverse bone outcomes in treated hypothyroidism, although the role of undertreatment, if any, is less clear.

### Neurocognitive outcomes

Overt (untreated) hypothyroidism has well established negative cognitive effects, including deficits in verbal memory, attention, language, psychomotor function, and executive function (48–51). There is some evidence that subtle detrimental effects on cognition may not be fully reversed with thyroid hormone replacement (21, 52). Several studies utilizing a variety of measurement tools to evaluate neurocognitive function have demonstrated persistent deficits in some but not all measurements following treatment (48, 53, 54). In particular, in a cross-sectional, interventional study, spatial and associative memory deficits were shown to persist despite treatment in overt hypothyroidism, implicating the hippocampus as a brain region that is particularly sensitive to low thyroid hormone levels (49). However, this study did not provide specific TSH levels following treatment, allowing for the possibility of undertreatment or overtreatment at the time of cognitive testing. The underlying mechanisms of persistent deficits in cognitive function are not well understood, however neuropathological studies have pointed to cerebrovascular disease being a more likely culprit compared to neurodegeneration (55). A cross-sectional functional MRI (fMRI) study of younger individuals with Hashimoto’s disease on treatment with normalized TSH levels (mean age, 32 years; mean treatment duration 4.4 years; mean TSH 2.0 mIU/L) did not reveal cognitive deficits in a battery of neuropsychological tests performed during fMRI acquisition (56). However, the duration of treatment was associated with differences in grey matter in the supracalcarine cortex, inferior frontal gyrus, amygdala, and frontal cortex. The exact ramifications of these changes are not yet clear. Additional studies utilizing fMRI and PET techniques have implicated glucose metabolism and cerebral blood flow as potential mediators of the negative effects of hypothyroidism on cognitive function (57–59), although data in treated individuals are sparse.

Not all studies have identified cognitive deficits in treated individuals. A cross-sectional study of mostly older women with an average of 20 years on treatment with normal TSH levels (mean age 76.1 years; mean TSH 1.54 mIU/L) showed similar cognitive functioning results compared to euthyroid peers (mean age 73.6 years) (60). Several studies of overtly hypothyroid patients have demonstrated significant pretreatment cognitive deficits, which normalized after treatment (61, 62). Two studies have also examined the relationship between changes in TSH within the reference range and cognitive outcomes, both of which found no association between TSH level and cognitive function (63, 64).

**Figure 1:**
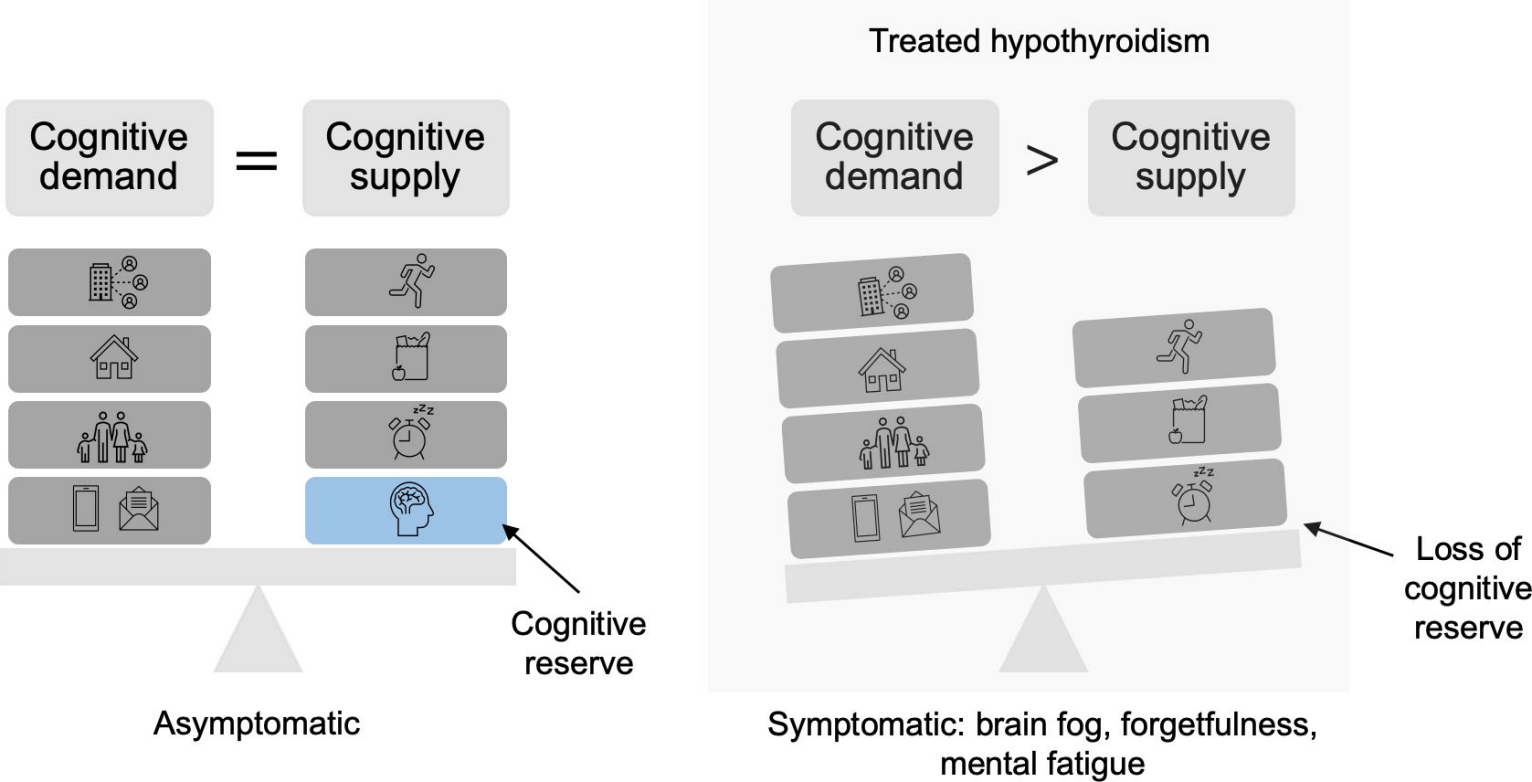
Depiction of loss of cognitive reserve in treated hypothyroidism. Cognitive demands consist of the daily responsibilities that require attention. Cognitive supply is supported by positive habits, including adequate nutrition, exercise, and rest. When full neurocognitive function due to overt hypothyroidism is not fully restored, which can occur with thyroid hormone replacement, cognitive reserve is limited. As a result, cognitive demand outweighs cognitive supply, causing symptoms.

A potential rationale for the conflicting data on cognitive outcomes in treated hypothyroidism may be that each individual patient has a set “cognitive demand” that is determined by mental tasks that vary in intensity daily, and a “cognitive reserve” to compensate if thyroid hormone treatment does not fully restore normal neurocognitive function on a cellular and tissue basis within the brain (**Figure 1**). It is possible that symptoms arise when demand is greater than supply, which for some patients may occur frequently for some but rarely for others (56, 65).

### Inherent limitations of health outcome studies

Studies that examine the association between degree of treatment of hypothyroidism (i.e. overtreatment and undertreatment) and health outcomes from real-world datasets provide a measure of the effectiveness of thyroid hormone replacement and also demonstrate the consequences of overtreatment and undertreatment. However, there are several important limitations that must be considered when interpreting these types of studies. First, there is a degree of selection bias built into these studies because patients without thyroid function tests are often not included. In a study of an adult German population with either a diagnosis of thyroid disease or a prescription for thyroid hormone, up to 40% of the population did not have an available TSH value over a 12 month period (36). It is likely that by studying only patients with available thyroid function studies, a substantial proportion of the hypothyroid population and therefore outcome events remain uncaptured. Second, as many studies seek to retrospectively determine outcomes of the treatment of hypothyroidism, often data on whether patients were initially treated for overt or subclinical hypothyroidism are not available. Outcomes may differ between these groups due to differences in endogenous thyroid hormone production, dose requirements, presence of antibodies, etc. It is important to keep in mind that these studies seek to determine the outcome of a real-world health care intervention with the understanding that thyroid hormone is prescribed to resolve biochemical abnormalities and/or symptoms in both overt and subclinical hypothyroidism.

Third, it is likely that inconsistent use of thyroid hormone coincides with inconsistent use of other medications that modify cardiovascular risk. While isolating the specific effect of thyroid hormone on a clinical outcome remains a challenge in study design and statistical approach, TSH level remains an important marker of care for an individual patient. It can be reasonably assumed that patients with a strong pattern of adherence to thyroid hormone also regularly take other appropriate therapies, and vice versa. Fourth, there is no consistent definition of overtreatment and undertreatment of thyroid hormone in the existing literature with respect to which thyroid function tests should be included, what levels should be considered “abnormal,” and over what time period. The clinical use of individualized TSH reference ranges remains a point of heated debate (66). Comprehensive, longitudinal studies of serial thyroid function tests and clinical outcomes may provide valuable insight for clinicians as to realistic treatment goals for patients with hypothyroidism to minimize adverse outcomes.

## Patient-reported outcomes

### ThyPRO

The Centers of Medicare and Medicaid Services define a patient reported outcome (PRO) as any report of a patient’s health condition that is provided by the patient without interpretation of the response by a researcher or health care provider (67). Over a decade ago, researchers in Denmark developed a thyroid-specific patient reported outcome (ThyPRO) questionnaire (68, 69), which has subsequently been validated in a variety of settings to measure QoL in people with thyroid disease (70). ThyPRO was designed to evaluate three primary categories of PROs: 1) physical symptoms, 2) tiredness and mental health, and 3) impact of thyroid disease on daily life. The current version of the ThyPRO questionnaire has 85 items and is reported in multiple scales. A shorter version (ThyPRO-39) has been developed by the original authors, which consolidates questions to address physical symptoms, mental and social well-being, appearance, and overall QoL (71). A standard measurement tool across patient populations has proved beneficial in a variety of research settings, including measuring the effect of levothyroxine therapy on QoL in subclinical hypothyroidism (72). The role of ThyPRO in the clinic setting is less clear, although researchers have sought to determine the minimal important change in ThyPRO scoring should it be used in a serial fashion over the course of treatment (73). Since its development, the extent to which ThyPRO has been used in the real-world clinic setting—either in primary care or endocrinology clinics—as a measure of the effectiveness of thyroid hormone treatment has not been described.

### PROs in treated hypothyroidism

Since the development of the ThyPRO (69, 71) and the hypothyroidism-specific ThyDQoL (74) questionnaires, several studies have examined PROs in those with hypothyroidism. Importantly, any PRO measurement must be interpreted within the context of the patient’s thyroid function at the time of assessment. For example, a high fatigue score in the setting of an elevated TSH level would first be thought to be due to undertreatment with thyroid hormone. The fatigue score simply reflects how the patient feels at that time, regardless of the cause of those symptoms. The result remains a true reflection of the patient’s experience (for an individual or a population), however the corresponding thyroid function test would direct the clinician as to how to approach those symptoms. An abnormal thyroid function test may trigger a thyroid hormone dose adjustment. It is equally important to recognize that persistent hypothyroid symptoms in an individual patient in the setting of normal thyroid function may not be the result of suboptimal thyroid replacement, but due to another cause.

How do QoL scores of treated subjects compare to controls without hypothyroidism? Numerous randomized and observational studies have included QoL measures as primary or secondary outcomes in patients with treated clinical and subclinical hypothyroidism (75), with many utilizing ThyPRO as a primary or secondary outcome measure (22, 64, 76–80). Overall, the body of literature is quite heterogeneous (e.g., population demographics, etiology of hypothyroidism), and many compare different types of treatment or treatment versus no treatment in the case of subclinical hypothyroidism. As a result, the literature does not lend itself to any generalized conclusions on PROs in treated hypothyroidism. There are studies comparing PROs in treated hypothyroidism versus healthy controls that suggest that some deficits in mood and cognitive QoL parameters may persist in some participants despite normalization of TSH and thyroid hormone levels (22, 81, 82). Adequate and suppressive treatment of hypothyroidism have been associated with worse QoL scores compared to healthy controls (81). Thyroid autoimmunity appears to also play a role (22, 82). One prospective study of 78 patients with diagnosed chronic autoimmune (Hashimoto’s) thyroiditis were followed from the point of diagnosis to 6 months after the onset of treatment with levothyroxine (22). ThyPRO scores improved from baseline after treatment, but moderate differences between the healthy control population remained. Of note, nearly 30% of subjects had a moderately elevated TSH at the time of PRO assessment. While undertreatment could account for lower QoL scores, additional analysis did not identify a relationship between TSH level and QoL score amongst the treated study participants. Similar results have been seen in other studies that have included patients with autoimmune thyroid disease (23, 78, 83). Of note, a post-thyroidectomy study found restoration of QoL scores similar to that of a healthy control population after treatment for nontoxic nodular goiter, although many patients treated with subtotal thyroidectomy did not require thyroid hormone after surgery (84).

### The limitations of using PRO studies to inform clinical management

A reasonable definition of successful treatment of hypothyroidism should include the restoration of thyroid function (defined by biochemical tests) and resolution of symptoms (measured formally by PROs). This aligns with prominent society guidelines on the treatment of hypothyroidism, which outline three primary goals of treatment: 1) normalization of TSH; 2) resolution of symptoms; 3) avoiding overtreatment (85, 86). However, as observed in several of the aforementioned studies examining QoL in treated hypothyroidism, normalization of TSH level does not always guarantee “normalization” of QoL for some patients. Furthermore, based on multiple clinical trials, changes in TSH within the normal and near-normal reference range do not appear to affect QoL scores for most patients (64, 87). A cross-sectional study of 218 patients taking thyroid hormone and with normal TSH levels using ThyPRO found that only the tiredness scores increased (i.e., more tiredness) with increasing TSH, while all other QoL domains were not correlated with TSH level (80). A reasonable interpretation of these results, given the non-specific nature of hypothyroid symptoms, is that lower QoL scores in treated patients with normal TSH levels are the result of other conditions or lifestyle behaviors, as opposed to undertreated hypothyroidism. In QoL studies that include patients treated for subclinical hypothyroidism, there is likely some degree of selection bias due to the presence of non-specific symptoms being the reason to screen for hypothyroidism and treat in the first place. We caution against interpreting the PRO literature as evidence that TSH should not guide treatment, or that patients with persistent symptoms should be “overtreated.” On the contrary, the body of literature of outcomes research reviewed previously has provided strong evidence that overtreatment with a suppressed TSH should be avoided as much as possible. We advocate for guideline-directed use of levothyroxine only in the setting of a confirmed diagnosis of hypothyroidism or in certain cases of subclinical hypothyroidism (88), with a TSH treatment target within the normal range. Persistent symptoms despite normalization of TSH should be investigated by the physician, and other underlying causes should be addressed. Some experts argue for a trial of T4 and T3 (i.e. combination therapy) in patients with persistent symptoms, as some patients may benefit from T3 (89). While a comprehensive discussion of the use of combination therapy is outside of the scope of this review, there is general agreement that further clinical trials are needed to support its use on a routine basis.

**Figure 2:**
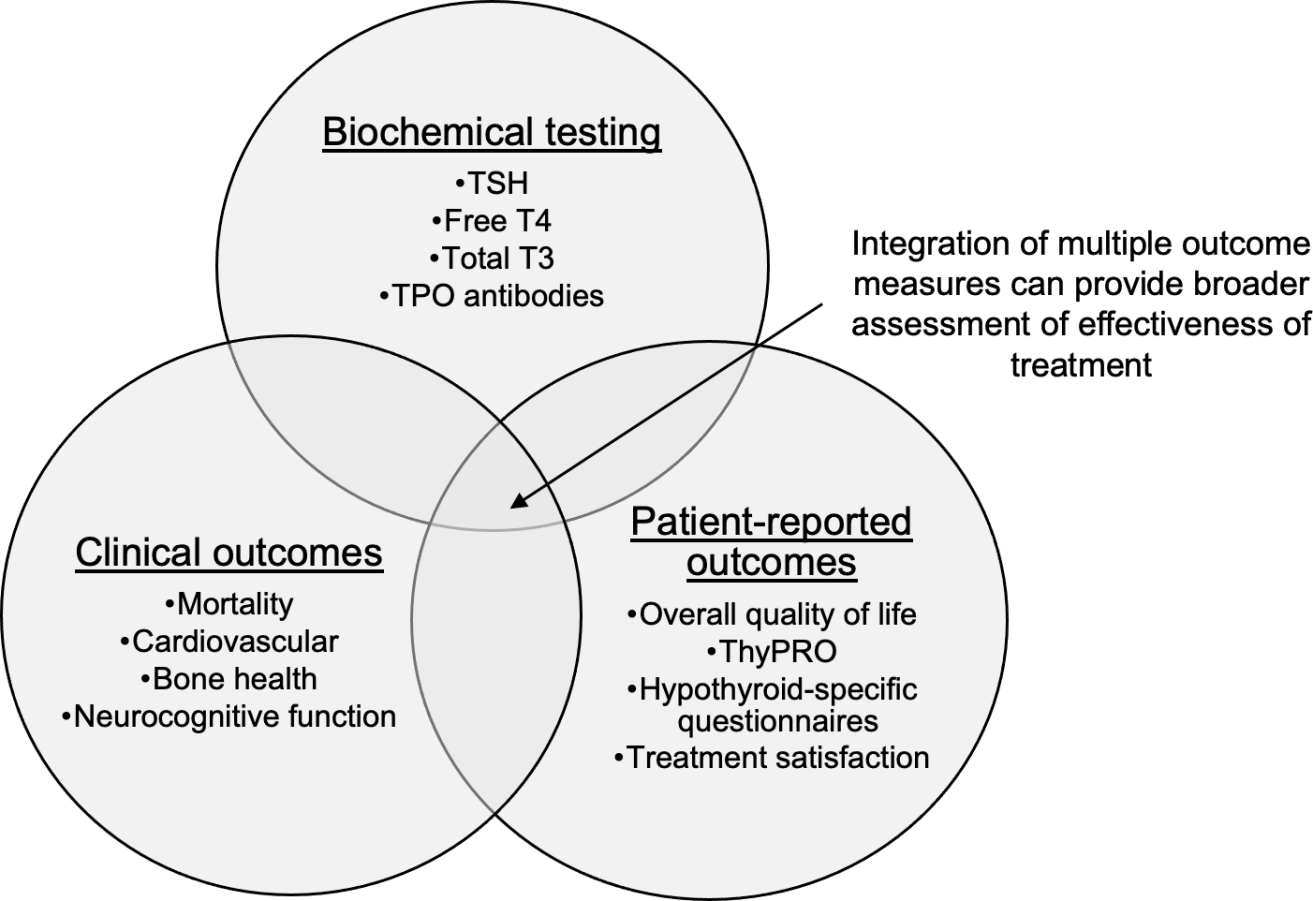
Intersection of health outcomes hypothyroidism research. TSH, thyrotropin; T4, thyroxine; T3, triiodothyronine; TPO, thyroid peroxidase; ThyPRO, thyroid specific patient reported outcome questionnaire.

Most outcomes studies have focused on key clinical outcomes (e.g., mortality or cardiovascular morbidity) or PROs, but not both. Many studies consider thyroid function testing in the context of intensity of treatment (i.e., overtreatment, undertreatment, or appropriate treatment). However, there may be a role for questionnaires such as ThyPRO to be used not as a study outcome but as a measurement of treatment, along with thyroid function testing. For example, such a study design could explore health outcomes in those with normal thyroid function but persistent symptoms or lower QoL scores. Of course, these types of studies must overcome limitations such as confounding factors and would require long-term survey follow up. However, examination of the relationship between hypothyroidism treatment and clinical health outcomes, in the context of both PROs and biochemical thyroid function (**Figure 2**), may lead to a more nuanced and useful definition of the successful treatment of hypothyroidism.

## Sociodemographic disparities in clinical outcomes

To date, there has been relatively few investigations into inequalities in the treatment of benign thyroid disease between sociodemographic groups, although more attention has been paid to the issue in recent years (90). When interpreting disparities research in hypothyroidism, it is important to recognize that disparities in the initiation of thyroid hormone have been identified in the background of increasing thyroid hormone prescriptions in the general population and across sociodemographic groups (91, 92). While untreated hypothyroidism has become increasingly less common, there is evidence that untreated hypothyroidism remains more likely for certain patient groups, in particular for men, individuals younger than 45 years of age, and those without access to regular health care (93, 94). Several studies have examined racial/ethnic factors that influence whether a patient will receive treatment for hypothyroidism (95–99). An analysis of the Cardiovascular Health Study dataset of those aged 65 years and older demonstrated that White women over any other race and sex group were more likely to start treatment with thyroid hormone (96). A study of the Baltimore Longitudinal Study of Aging found similar results (97). A study of the Canadian Community Health Survey found that non-White individuals were less likely to be screened for thyroid disease, but found no differences in screening or treatment between men and women (98). Lastly, among pregnant women in the U.S. with subclinical hypothyroidism, Hispanic women were less likely than White women to receive levothyroxine (99). Overall, it appears that age, sex, and race/ethnicity influence the likelihood of an individual receiving thyroid hormone. Whether differences exist in the clinical outcomes of treated hypothyroidism between different sociodemographic groups remains unclear.

Overall, there are several challenges that limit our ability to identify sociodemographic disparities in clinical outcomes in treated hypothyroidism. The interplay between age, sex, race/ethnicity, and thyroid function, and their relationship between clinical outcomes, is made more complex by potential differences in the “normal” ranges of thyroid function between different sociodemographic groups (100). Furthermore, a large body of investigative work has been dedicated to defining and measuring sociodemographic and racial/ethnic disparities in clinical outcomes relevant to hypothyroidism, including cardiovascular morbidity/mortality and bone health (101–103). While there is no expectation that changes in thyroid hormone prescribing alone would ameliorate these disparities, any future studies examining sociodemographic disparities in clinical outcomes in the hypothyroid population would have to be interpreted within the context of these baseline differences.

## Knowledge gaps and future directions

As mentioned previously, significant knowledge gaps remain in the study of health outcomes in treated hypothyroidism. In several of the large, retrospective studies, delineating between outcomes of treatment for overt versus subclinical hypothyroidism is not possible. Studies identifying patients at the time of diagnosis and initiation of treatment and measuring outcomes in a prospective manner would allow investigators to distinguish outcomes between these two groups. Furthermore, stratifying study populations not only by diagnosis, but also by age, sex, race/ethnicity, geographic location, or access to health care could further develop our limited knowledge of disparities in treatment (90). Our understanding of the clinical consequences of overtreatment and undertreatment has been solidified over the last 10-15 years, but the questions of “who” is at risk of suboptimal treatment, and “why,” remain largely unanswered. Finally, one of the principal challenges in the management of hypothyroidism remains the patient with normal thyroid function and persistent hypothyroid symptoms. What is the role of outcomes research in helping to address this challenge? PROs can help identify those patients with persistent symptoms, but the evidence does not support using PRO/QoL measures to guide therapy. From clinical experience, we believe successful treatment of hypothyroidism in challenging cases often requires a multi-faceted approach, centered on an appropriate level of thyroid hormone replacement to achieve normalized thyroid function, with appropriate management of comorbid conditions, lifestyle adjustments, and a positive patient-physician relationship. Utilizing defined biochemical, clinical, and patient-reported outcomes as measuring posts, outcomes research can provide future insight on which health care practices in hypothyroidism provide measurable benefit to patients, and how to implement such practices.

## Conclusion

The study of health outcomes in treated hypothyroidism has yielded important findings that have expanded our understanding of the effectiveness of thyroid hormone replacement to restore normal thyroid function, improve QoL measures, and prevent adverse outcomes associated with hypothyroidism. Numerous studies have been conducted examining health outcomes or PROs in the context of TSH and thyroid hormone levels. Overall, these studies have demonstrated that there is room for improvement in the treatment of hypothyroidism with respect to health outcomes and QoL measures, and that maintaining thyroid function within the normal range for the duration of the treatment course is paramount to minimizing adverse health outcomes. Generally, restored thyroid function levels are associated with normalized cardiovascular health outcomes, but periods of overtreatment and undertreatment, if not rapidly corrected, increase the risk of adverse cardiovascular outcomes and overall mortality. Some deficits in cognitive function have been shown to persist even in the setting of normalized thyroid function. PROs in treated hypothyroidism have tended to follow a similar pattern in which lower QoL scores have persisted despite normalized thyroid function, particularly in populations with underlying autoimmune thyroid disease. While these thyroid function-outcome disparities warrant further investigation, there is no evidence to support titrating thyroid hormone treatment to resolve hypothyroid symptoms at the expense of normal thyroid function levels. Significant knowledge gaps also remain in the prevalence and underlying causes of sociodemographic disparities in health outcomes and PROs in hypothyroidism. Future prospective studies which integrate multiple health and patient-reported outcomes, along with biochemical thyroid testing, would provide a better understanding of the quality gaps—and how to improve them—for many patients with hypothyroidism.

## Author contributions

ME generated the initial outline, completed the literature review and authored the manuscript. MP reviewed the outline and authored the manuscript. All authors contributed to the article and approved the submitted version.

## Funding

The study was supported by the National Institute of Diabetes and Kidney Disease (NIDDK) of the National Institutes of Health (NIH) under award 5T32DK007011-46 ME. MP is supported by the National Institute on Aging (NIA) under award K08AG049684.

## Conflict of interest

The authors declare that the research was conducted in the absence of any commercial or financial relationships that could be construed as a potential conflict of interest.

The handling editor JJ declared a past co-authorship with the author ME.

## Publisher’s note

All claims expressed in this article are solely those of the authors and do not necessarily represent those of their affiliated organizations, or those of the publisher, the editors and the reviewers. Any product that may be evaluated in this article, or claim that may be made by its manufacturer, is not guaranteed or endorsed by the publisher.
